# Morbidity and Outcomes of Foreign Travelers in Zakynthos Island, Greece: A Retrospective Study

**DOI:** 10.1371/journal.pone.0094416

**Published:** 2014-04-11

**Authors:** Eleni Plessa, Giannoula S. Tansarli, Dimitrios Xanthopoulos, Matthew E. Falagas

**Affiliations:** 1 Zante Medical Care (ZanteMed), Zakynthos, Greece; 2 Alfa Institute of Biomedical Sciences (AIBS), Athens, Greece; 3 Department of Internal Medicine - Infectious Diseases, Iaso Hospital, Iaso Group, Athens, Greece; 4 Department of Medicine, Tufts University School of Medicine, Boston, Massachusetts, United States of America; The George Washington University Medical Center, United States of America

## Abstract

**Background:**

Although there is satisfactory recording of diseases affecting travelers visiting developing countries, little is known regarding morbidity of travelers when visiting developed countries. We sought to evaluate the morbidity of foreign travelers in Zakynthos, a popular Greek island attracting large number of foreign tourists every summer.

**Methods:**

Data from foreign travelers that accommodated in Zakynthos and sought medical services from the private offices of Zante Medical Care from May 1 to October 30 2012 were retrospectively analyzed.

**Results:**

Two thousand six hundred and eighty-eight patients were included in the study. The mean age (±SD) of the patients whom the age was recorded was 29.6 (±18.3) and 51.5% of them were from 18 to 40 years old. Disorders of the respiratory tract (32.7%), dermatologic conditions (21.1%), musculoskeletal injuries (16.4%), and gastrointestinal disorders (16.3%) were the four most prevalent clinical categories among patients. Ear disorder was the most common syndromic description (14.5%) among which 81.2% were ear infections; otitis externa and otitis media were diagnosed in 8.5% and 3.3% patients in total. The most common specific diagnosis was gastroenteritis (14.3%). Insect bite and sunburn were the most common diagnosis (6.5% and 3.8%, respectively) among patients with a dermatologic condition. Ear infection was the most common diagnosis in pediatric patients.

**Conclusion:**

Disorders mainly of the upper respiratory tract were the predominant causes of illness among foreign travelers in Zakynthos. Traveler's diarrhea was the most common specific diagnosis but the prevalence within the total population was not very high.

## Introduction

Travel medicine is routinely associated with diseases occurring among international travelers from developed countries when visiting developing countries. Traveler's diarrhea is the most commonly encountered disease among them. However, little is known regarding the diseases affecting travelers in developed regions of the world such as Europe,[1], [2] even though Europe traditionally receives more than 50% of the world's tourist arrivals.[3]

Greece is one of the world's most popular European tourist destinations,[4] especially in summer, attracting travelers from all over the world. The first study describing the morbidity of foreign travelers in Greece was published in 2012 and reported on travelers who accommodated in Attica and sought medical services from SOS Doctors, a network of physicians who perform house-call visits.[5] The study showed that the diseases of the respiratory tract were the most common disorders during all seasons. However, the islands of the Aegean and Ionian Sea are the most popular choices among tourists visiting Greece for summer vacations and therefore, it would be interesting to investigate the morbidity of these travelers while on vacation in a Greek island.

In this context, we aimed to outline the morbidity of foreign travelers in Zakynthos which is a popular Greek island attracting large number of foreign tourists every summer.

## Methods

### Study design, setting and patient population

This is a retrospective study evaluating data from foreign travelers that accommodated in Zakynthos island in the Ionian Sea, Greece, and sought medical services from the Zante Medical Care during a 6-month period, from May 1 to October 30 2012.

Zante Medical Care is a network of physicians that provides outpatient services in Zakynthos island.[6] Five offices with specialized medical and nursing staff are located at the most crowded tourist resorts of the island. There is one public hospital in Zakynthos which is not very easily accessible by foreign travelers due to its location, while there are no private hospitals. Finally, there is another private medical company with similar profile to Zante Medical Care and 3 independent general practitioners whom offices are near tourist resorts.

Patients with incomplete medical record (i.e. due to early departure), inconsistent follow-up, and non-compliance to doctor's recommendations (refusal to undertake laboratory or imaging tests which were considered necessary by the doctor) were excluded from the study.

### Ethics considerations

A written or oral informed consent was obtained neither by the adult nor the minors/children participants because of the non-interventional retrospective design of the study. This was waived by the approving Institutional Review Board of Zante Medical Care. The study was approved by the Research Ethics Committee of Zante Medical Care.

### Data collection

The reviewed data of the included patients comprised the following: age, gender, nationality, month of visit, duration of travel, medical diagnosis, clinical category of the diagnosis, laboratory and imaging tests performed, day observation, and need for hospitalization. The laboratory or imaging tests reported on the patients who performed the tests in the outpatient setting at the request of the physicians of the Zante Medical Care but not on those who performed these tests in the hospital. Day observation was defined as the patients' support with intravenous fluids or inhalers.

All medical diagnoses were stratified into one of the following general clinical categories: respiratory (both upper and lower) disorders, dermatologic conditions, musculoskeletal injuries, gastrointestinal disorders, genitourinary disorders, ophthalmologic disorders, non-specific symptoms, cardiovascular disorders, systemic febrile illness, gynecologic-obstetric disorders, dental problems, neurologic disorders, psychiatric disorders, chronic disease, drug-related adverse events, and other conditions (including patients contacting the physicians of Zante MedCare for receiving a medical certification). Pediatric patients were defined those being younger than 18 years old.

### Data analysis

Firstly, the relative frequencies of the most common specific diagnoses within each clinical category and the proportionate morbidity by clinical category of the specific diagnoses were calculated. The percentage of patients who needed day observation or hospitalization within each clinical category was also calculated. Then, the proportionate morbidity was analyzed according to, age, gender, nationality, month of visit, and duration of travel. Finally, univariate analyses were performed with regard to day observation and need for hospitalization. Chi-square or Fisher's exact test was used for the comparison of categorical variables. All variables were also entered into a binary logistic regression model, regardless the significance in the univariate analysis. The adjusted odds ratio (OR) and confidence intervals (CIs) were calculated. The SPSS 17.0 software (SPSS Inc., Chicago, IL, USA) and the Openepi[7] were used for the statistical analyses. A p-value <0.05 was considered to indicate statistical significance.

## Results

### Demographic characteristics

Three thousand two hundred foreign travelers accommodated in Zakynthos and sought medical services from Zante Medical Care during the study period. Among those, two thousand six hundred and eighty-eight patients were included in this study; the remaining patients were excluded because they denied undertaking the necessary imaging tests or did not show up at an arranged follow-up visit. The vast majority of the included patients were tourists. The mean age (±SD) of the patients whom the age was recorded was 29.6 (±18.3) and 78.5% of them were adults. The majority of patients (51.5%) were from 18 to 40 years old and 57% were females. British, Serbian and Hungarian were the nationalities with the highest prevalence (27.5%, 21.8% and 17.5%, respectively). The demographic characteristics of the included patients are presented in Table 1.

**Table 1:** Demographic characteristics of 2,688 patients.

Characteristic	*n* (%)
Age (y), mean ± SD	29.6±18.3
Age group (y)*	
0–5	221 (8.2)
6–11	186 (6.9)
12–17	150 (5.6)
18–40	1,385 (51.5)
40–60	436 (16.2)
>60	217 (8.1)
NR	92 (3.4)
Gender (F)*	1,533 (57)
Country	
United Kingdom	740 (27.5)
Serbia	586 (21.8)
Hungary	471 (17.5)
Russia	155 (5.8)
Poland	138 (5.1)
Denmark	110 (4)
Czech Republic	97 (3.6)
Netherlands	66 (2.5)
Romania	59 (2.2)
Other**	266 (9.9)

^*^Age was not recorded for 92 patients, while gender was not recorded for 7 patients.

^**^ The remaining travelers were from Italy (36), former Yugoslav Republic of Macedonia (30), Finland (29), Sweden (27), Slovenia (26), Ireland (24), Bulgaria (17), Austria (16), Belgium (10), Germany (10), Norway (10), Croatia (11), Switzerland (5), Slovakia (2), Lithuania (2), Australia (1), France (1), Ukraine (2), Brazil (1), Bosnia (1), while relevant data was not recorded from 5 travelers.

**Abbreviations**

SD: standard deviation, y: year, F: female, UK: United Kingdom, SRB: Serbia, HU: Hungary, OR: odds ratio, CI: confidence interval

### Proportionate morbidity by clinical category and specific diagnoses within each category

The four most prevalent clinical categories among the included patients were the following: disorders of the respiratory tract (879 patients; 32.7%), dermatologic condition (566 patients; 21.1%), musculoskeletal injury (440 patients; 16.4%), and gastrointestinal disorders (437 patients; 16.3%). Gastrointestinal disorders were divided into diarrheal (385 patients; 14.3%) and non-diarrheal (54 patients; 2%). In total, the most common specific diagnosis was gastroenteritis (385 patients), while the second most common diagnosis was ear infection (316 patients). These findings were verified in the adult population of the study. Finally, the most common syndromic description of all was ear disorder (389 patients). More details on the remaining clinical categories and specific diagnoses within each clinical category are presented in Table 2.

**Table 2:** Morbidity of 2,688 patients by clinical category and most common specific diagnosis within each category.

*Clinical category*	*n* (%)
**Respiratory tract**	**879 (32.7)**
Otitis externa	228 (8.5)
Tonsillitis	103 (3.8)
Pharyngitis	100 (3.7)
Common cold	96 (3.6)
Otitis media	88 (3.3)
Acute bronchitis	54 (2)
**Dermatologic**	**566 (21.1)**
Insect bite	174 (6.5)
Sunburn	101 (3.8)
Allergic rash	97 (3.6)
**Musculoskeletal injury**	**440 (16.4)**
Laceration	81 (3)
Fractures	77 (2.9)
Abrasion	71 (2.6)
Sprains and dislocations	53 (2)
**Diarrheal Gastrointestinal**	**437 (16.3)**
Gastroenteritis	385 (14.3)
**Genitourinary**	**75 (2.8)**
Cystitis	66 (2.5)
**Ophthalmologic**	**77 (2.9)**
Conjunctivitis	68 (2.5)
**Non-specific symptoms**	**65 (2.4)**
Lower extremity edema	18 (0.7)
Alcohol intoxication	8 (0.3)
**Non-diarrheal Gastrointestinal**	**54 (2)**
Dyspepsia	14 (0.5)
**Cardiovascular**	**31 (1.2)**
Hypertension	10 (0.4)
**Systemic febrile illness**	**15 (0.6)**
**Gynecologic/Obstetric**	**17 (0.6)**
**Dental**	**12 (0.4)**
**Neurologic**	**12 (0.4)**
**Psychiatric**	**10 (0.4)**
**Chronic disease**	**16 (0.6)**
**Drug-related adverse events**	**6 (0.2)**
**Other**	**26 (1)**

**Abbreviations**

SD: standard deviation, y: year, F: female, UK: United Kingdom, SRB: Serbia, HU: Hungary, OR: odds ratio, CI: confidence interval.

Forty-two patients performed laboratory tests namely blood test (21 patients), urine culture (13), ear drainage culture (9). One patient performed both blood test and urine culture. One hundred and fifty-two patients performed imaging tests namely X-ray ankle (36), X-ray chest (21), X-ray foot (20), X-ray wrist (12), X-ray knee (10), and other (53).

#### Pediatric population

Five hundred and ninety-eight out of 2,688 patients (22.2%) were pediatric. Disorders of the respiratory tract were the most common among this patient population (277 patients; 46.3%). One hundred and two (17.1%) and one hundred and four (17.4%) patients had a gastrointestinal and dermatologic disorder, respectively. Sixty-seven patients (11.2%) had musculoskeletal injury, twelve patients (2%) had systemic febrile illness, twelve patients (2%) had ophthalmologic disorder, and 24 patients (8%) had another disorder including non-specific symptoms and disorders of the genitourinary tract. With regard to specific diagnoses, ear infection was the most common diagnosis (117 patients; 19.6%) followed by gastroenteritis (66 patients; 14.2%). Ear infection included otitis externa (65 patients; 10.9%) and otitis media (52 patients; 8.7%), while rhinopharyngitis was diagnosed in 39 patients (6.5%) and tonsillitis in 29 patients (4.8%).

### Proportionate morbidity according to age, gender, nationality, month of visit, duration of travel

In Table 3 the proportionate morbidity of each clinical category is described according to age (< or ≥18 years), gender, month of visit, and duration of travel (<10 days, 10–30 days, >30 days). In particular, disorders of the respiratory tract were relatively more frequent (p<0.001) among patients younger than 18 years old compared to adults (47.9% versus 28.6%), among males compared to females (36.8% versus 29.5%), and among Serbians compared to British or Hungarian tourists (38.2% versus 28.8% or 21.9%, respectively). Additionally, these diseases occurred relatively more frequently (p<0.001) in August than in July, May, September, June, and October (39.4% versus 34%, 29.3%, 28.7%, 27.2%, and 23%, respectively) and among patients who stayed from 10 to 30 days in the island compared to those who stayed for >30 days or for <10 days (39.9% versus 33.3% or 27.6%, respectively).

**Table 3:** Morbidity and univariate analysis of 2,688 patients according to age, gender, month of travel, and duration of stay.

Clinical category	Age (y)*		p-value	Gender*		p-value	Country				p-value	Month of visit						p-value	Duration of travel (d)*			p-value
	<18 (557)	≥18 (2038)		M (1148)	F (1533)		UK (740)	SRB (586)	HU (471)	Other (891)		May (133)	June (489)	July (874)	Aug (680)	Sept (425)	Oct (87)		<10 (1451)	10–30 (1046)	>30 (54)	
Respiratory tract	267 47.9%	582 28.6%	<0.001	422 36.8%	453 29.5%	<0.001	213 28.8%	224 38.2%	103 21.9%	339 38.1%	<0.001	39 29.3%	133 27.2%	297 34%	268 39.4%	122 28.7%	20 23%	<0.001	400 27.6%	417 39.9%	18 33.3%	<0.001
Dermatologic	92 16.5%	450 22.1%	0.02	213 18.6%	353 23%	0.005	187 25.3%	154 26.3%	84 17.8%	141 15.8%	<0.001	32 24.1%	113 23.1%	194 22.2%	135 19.9%	79 18.6%	13 14.9%	0.24	300 20.7%	224 21.4%	8 14.8%	0.52
Musculoskeletal injury	55 9.9%	373 18.3%	<0.001	220 19.2%	218 14.2%	0.001	141 19.1%	74 12.6%	61 13%	164 18.4%	0.001	31 23.3%	75 15.3%	132 15.1%	97 14.3%	83 19.5%	22 25.3%	0.006	255 17.6%	150 14.3%	12 22.2%	0.002
Gastrointestinal	97 17.4%	332 16.3%	0.62	157 13.7%	281 18.3%	0.001	74 10%	70 11.9%	170 36.1%	125 14%	<0.001	12 9%	91 18.6%	145 16.6%	96 14.1%	78 18.4%	17 19.5%	0.04	306 21.1%	114 10.9%	5 9.3%	<0.001

^*^ Data on age, gender, and duration of travel was not available for 92, 7, and 137 patients, respectively.

**Abbreviations**

y: year, F: female, M: male, d: day, UK: United Kingdom, SRB: Serbia, HU: Hungary, OR: odds ratio, CI: confidence interval, Aug: August, Sept: September, October.

Dermatologic conditions were more relatively in adults than patients younger than 18 years old (22.1% versus 16.5%, p = 0.02), in females than males (23% versus 18.6%, p = 0.005), and in Serbians and British than Hungarian tourists (26.3% and 25.3%, respectively, versus 17.8%, p<0.001).

Musculoskeletal injury was relatively more frequent (p<0.001) in adults than patients younger than 18 years old (18.3% versus 9.9%), in males than females (19.2% versus 14.2%), and in British than Hungarian or Serbian tourists (19.1% versus 13% or 12.6%, respectively, p = 0.001). In addition, they were relatively more frequent in October than in May, September, June, July, and August (25.3% versus 23.3%, 19.5%, 15.3%, 15.1%, and 14.3%, respectively, p = 0.006) and in those who stayed for more than 30 days in the island than those who stayed for less than 10 days or from 10 to 30 days (22.2% versus 17.6% or 14.3%, respectively).

Last, disorders of the gastrointestinal tract were relatively more frequent in females than in males (18.3% versus 13.7%, p<0.001), in Hungarian tourists than in Serbians or British (36.% versus 11.9% or 10%, respectively, p<0.001), in October than in June, September, July, August, and May (19.5% versus 18.6%, 18.4%, 16.6%, 14.1%, and 9%, respectively, p = 0.04), and among those who stayed in the island for less than 10 days compared to those who stayed from 10 to 30 days or for more than 30 days (21.1% versus 10.9% or 9.3%, p<0.001).

### Risk factors for respiratory disorders, dermatologic conditions, musculoskeletal injuries and gastrointestinal disorders

Binary logistic regression analyses were performed in order to identify the risk factors for the development of the 4 most common categories of disease. The results of the analyses are presented in detail in Table 4. In particular, patients of younger age (<18 years old) were more likely to acquire respiratory disorders than adults (OR: 2.77, 95% CI: 2.22–3.46), while females and patients who stayed in the island for less than 10 days were less likely to acquire a respiratory disease than males and those who stayed for more than 30 days, respectively, [(OR: 0.75, 95% CI: 0.62–0.9) and (OR: 052, 95% CI: 0.27–0.99), respectively]. Females were more likely to have a dermatologic condition than males (OR: 1.25, 95% CI: 1.03–1.53), while Hungarians less likely than others (OR: 0.28, 95% CI: 0.19–0.41). Patients younger than 18 years old and females were less likely to have musculoskeletal injuries than adults and males, respectively, [(OR: 0.49, 95% CI: 0.36–0.65) and (OR: 0.64, 95% CI: 0.51–0.79), respectively]. Finally, tourists who stayed in the island for less than 10 days were more likely to develop gastrointestinal disorders than those who stayed for more than 30 days (OR: 2.66, 95% CI: 1.03–6.82), while females and tourists who visited the island in May were less likely than males and those who visited the island in October, respectively, [(OR: 1.39, 95% CI: 1.11–1.73) and (OR: 0.39, 95% CI: 0.17–0.89), respectively].

**Table 4:** Multivariate analysis regarding the development of the 4 most common categories of disease in 2,688 patients.

Variable	Respiratory tract			Dermatologic			Musculoskeletal injury			Gastrointestinal		
	OR	95% CI	p-value	OR	95% CI	p-value	OR	95% CI	p-value	OR	95% CI	p-value
Age (y)*												
≥18												
<18	2.77	2.22–3.46	<0.001	0.79	0.61–1.00	0.05	0.49	0.36–0.65	<0.001	1.12	0.86–1.44	0.41
Gender*												
F												
M	0.75	0.62–0.9	0.002	1.25	1.03–1.53	0.03	0.64	0.51–0.79	<0.001	1.39	1.11–1.73	0.004
Country												
UK	1.37	1.05–1.79	0.02	0.9	0.7–1.14	0.38	1.29	1.01–1.64	0.04	0.62	0.48–0.8	<0.001
SRB	6.7	5.16–8.71	<0.001	1.24	0.97–1.59	0.09	0.13	0.08–0.21	<0.001	0.44	0.33–0.6	<0.001
HU	8.22	6.23–10.85	<0.001	0.28	0.19–0.41	<0.001	0.33	0.22–0.48	<0.001	0.4	0.29–0.57	<0.001
Other	…	…	<0.001	…	…	<0.001	…	…	<0.001	…	…	<0.001
Month of visit												
May	1.27	0.63–2.56	0.51	2.06	0.98–4.31	0.06	1.01	0.51–2.00	0.99	0.39	0.17–0.89	0.03
June	0.99	0.54–1.82	0.98	1.78	0.92–3.44	0.09	0.66	0.36–1.18	0.16	1.00	0.55–1.82	0.99
July	1.29	0.72–2.3	0.4	1.8	0.95–3.42	0.07	0.66	0.37–1.16	0.15	0.94	0.53–1.68	0.84
Aug	1.75	0.97–3.14	0.06	1.44	0.75–2.76	0.27	0.63	0.36–1.13	0.12	0.74	0.41–1.33	0.31
Sept	1.28	0.69–2.34	0.43	1.37	0.7–2.67	0.36	0.82	0.46–1.48	0.52	0.95	0.52–1.73	0.86
Oct	…	…	0.005	…	…	0.11	…	…	0.21	…	…	0.05
Duration of travel (d)*												
<10	0.52	0.27–0.99	0.04	1.63	0.75–3.54	0.21	0.98	0.49–1.96	0.96	2.66	1.03–6.82	0.04
10–30	0.88	0.46–1.67	0.7	1.76	0.81–3.8	0.15	0.73	0.37–1.46	0.37	1.52	0.59–3.91	0.39
>30	…	…	<0.001	…	…	0.31	…	…	0.03	…	…	<0.001

^*^ Data on age, gender, and duration of travel was not available for 92, 7, and 137 patients, respectively.

**Abbreviations**

y: year, F: female, d: day, UK: United Kingdom, SRB: Serbia, HU: Hungary, OR: odds ratio, CI: confidence interval, Aug: August, Sept: September, October.

### Observation - Need for hospitalization

The proportions of patients who received day observation or needed hospitalization in each clinical category are presented in Table 3. Day observation had the highest prevalence (22.2%) among patients with gastrointestinal disease. Patients with respiratory tract disease had the highest occurrence (23.1%) of hospitalization among all clinical categories.

#### Risk factors

The results of the univariate analyses regarding observation and need for hospitalization are presented in Table 5. All variables that were compared in univariate analyses were entered into a binary logistic regression model. Adjustment for gender was performed in the analysis of observation and for gender and duration of travel in the analysis of need for hospitalization. The results of the analysis are presented in Table 4, as well. Age, gender, duration of travel, and clinical category of disease were all independently associated with observation. In particular, patients younger than 18 years old, females, and those who stayed for 10–30 days in the island were less likely to receive observation than adult patients, females, and those who stayed for less than 10 days in the island, respectively [(OR: 0.58, 95% CI: 0.37–0.89), (0.71, 0.53–0.96), and (0.73, 0.54–0.99), respectively]. Finally, patients with dermatologic condition or musculoskeletal injury or gastrointestinal disorder or a disorder not classified in any of these categories had higher odds to receive observation than patients who had a disorder of the respiratory tract [(OR: 3.25, 95% CI: 1.88–5.64), (2.36, 1.30–4.30), (4.39, 2.53–7.61), and (10.10, 6.03–17.05), respectively].

**Table 5:** Univariate and multivariate analyses regarding observation and need for hospitalization of 2,688 patients.

	Observation						Need for hospitalization					
			Univariate analyses	Multivariate analyses					Univariate analyses	Multivariate analyses		
Variable	Yes (n = 212)	No (n = 2,476)	p-value	OR	95% CI	p-value	Yes (n = 78)	No (n = 2,610)	p-value	OR	95%CI	p-value
Age (y)*												
≥18	178 (84)	1,818 (73.4)					51 (65.4)	1,945 (74.5)				
<18	26 (12.3)	572 (23.1)	<0.001	0.58	0.37–0.89	0.01	25 (32.1)	573 (22)	0.04	1.99	1.18–3.37	0.01
Gender												
F	117 (55.2)	1,416 (57.2)					40 (51.3)	1,493 (57.2)				
M	95 (44.8)	1,053 (42.5)	0.54	0.71	0.53–0.96	0.03	38 (48.7)	1,110 (42.5)	0.29	0.82	0.51–1.33	0.43
Duration of travel (d)*												
<10	103 (48.6)	971 (39.2)		…	…	0.04	30 (38.5)	1,044 (40)		…	…	0.77
10–30	92 (43.4)	1,331 (53.8)		0.73	0.54–0.99	0.04	43 (55.1)	1,380 (52.9)		1.09	0.67–1.78	0.72
>30	8 (3.8)	46 (1.9)	0.003	1.58	0.69–3.6	0.28	1 (1.3)	53 (2)	0.85	0.55	0.07–4.18	0.56
Category of disease												
Respiratory tract	20 (9.4)	859 (34.7)		…	…	<0.001	18 (23.1)	861 (33)		…	…	<0.001
Dermatologic	43 (20.3)	523 (21.1)		3.25	1.88–5.64	<0.001	0 (0)	566 (21.7)		0	0-.	0.99
Musculoskeletal injury	26 (12.3)	414 (16.7)		2.36	1.30–4.30	0.005	13 (16.7)	427 (16.4)		1.79	0.85–3.78	0.13
Gastrointestinal	47 (22.2)	392 (15.8)		4.39	2.53–7.61	<0.001	12 (15.4)	427 (16.4)		1.42	0.65–3.1	0.38
Other**	76 (35.8)	288 (11.6)	<0.001	10.14	6.03–17.05	<0.001	35 (44.9)	329 (12.6)	<0.001	6.25	3.36–11.64	<0.001

^*^ Data on age, gender, and duration of travel was not available for 92, 7, and 137 patients, respectively.

^**^ The remaining clinical categories are reported in Tables 2 and 3.

**Abbreviations**

y: year, F: female, d: day, UK: United Kingdom, SRB: Serbia, HU: Hungary, OR: odds ratio, CI: confidence interval.

Age and clinical category of disease were independently associated with need for hospitalization. More specifically, patients younger than 18 years old had double odds to need hospitalization compared to adult patients (OR: 1.99, 95% CI: 1.18–3.37), while the odds for patients with a disorder other than respiratory, dermatologic, musculoskeletal injury, or gastrointestinal were six times the odds for patients with respiratory disorder (OR: 6.25, 95% CI: 3.36–11.64).

## Discussion

Our study revealed that the disorders of the respiratory tract were the most commonly (32.7%) encountered among foreign travelers visiting Zakynthos during summer this finding is consistent with the findings of a previous study addressing the morbidity of foreign travelers visiting Attica, Greece.[5] Also, respiratory disorders were the leading causes of illness among European travelers who returned ill after a trip in Western[1] or Eastern Europe[2] in previous studies. Gastroenteritis was the most common specific diagnosis in the current study, while ear disorder was the predominant syndromic description.

Similarly to adult patients, disorders of the upper respiratory tract were the most common disorders and this finding was in consistently with a previous study showing that respiratory disorders were the most common disorders among pediatric patients after exposure to Europe and Northern America[15] In contrast to the findings of the current study on adult patients, ear infection and not gastroenteritis was the most common specific diagnosis among pediatric patients.

Respiratory disorders dominated among foreign travelers who visited Attica and called the SOS Doctors in previous studies[5], [10] and were observed more frequently in winter than the other seasons.[5] Exposure to cold has been associated with increase in the incidence of respiratory tract infections.[11], [12] The high prevalence of otitis externa in our study may be attributed to the wide use of swimming pools during summer, as acute otitis externa is a common problem among persons using swimming pools.[13], [14]

It is shown that Greece is a tourist destination where the prevalence of traveler's diarrhea is not very high. The prevalence of 14% for gastroenteritis, which was diagnosed during summer months in 75% of the cases, could be explained by the increased prevalence of food poisoning due to *Salmonella* and *Campylobacter* during hot months or by norovirus outbreaks that can be caused in overcrowded places during all year.[8] Avoiding food and water from places with questionable sanitary conditions might reduce to a minimum the occurrence of traveler's diarrhea in foreign travelers visiting Greece in summer. The higher prevalence of the gastrointestinal disorders (mostly gastroenteritis) in October than in other months, was probably due to high prevalence of gastrointestinal viruses during cooler months.[8] Insect bite with or without super-infection was the most common dermatologic condition in this study which is consistent with a previous study reporting on travelers returning from developing countries.[9] Additionally, the percentage of systemic febrile illness includes only viral syndromes of the childhood and was extremely low.

It was shown that male gender and age ≥18 years old were independently associated with day observation. In general, intravenous administration of fluids is needed more commonly in older ages than in younger. Also, early age (18 years old) was independently associated with hospitalization which is consistent with the findings of a previous study.[15] This may be because physicians tend to treat pediatric patients more conservatively than adult patients. Bronchiolitis, gastroenteritis, and head injury were the most common diagnoses among pediatric patients that were recommended for hospitalization.

The findings of our study should be interpreted considering certain limitations. First, the study design was retrospective and the data do not allow the estimation of the incidence of each clinical disorder and not on all travelers who arrived on the island had complete data; however, sufficient clinical information was available for analysis. Almost all studies on travel medicine are based on data collected from ill returned travelers which mainly concerns long-incubation diseases. The main advantage of the current study over the previous ones is the investigation of the morbidity of travelers while they were on vacation; therefore, the analyzed data focus on diseases with short incubation period and on injuries which has not been sufficiently described yet in the published literature. Furthermore, very few cases of alcohol intoxication were recorded because all 5 offices of Zante Medical Care were closed at night. Accordingly, the prevalence of alcohol intoxication in the current study certainly is not representative of its occurrence in the island. Yet, mild diseases may be over-represented in this study because patients with severe or life-threatening conditions were transferred directly to the hospital. Despite limitations, it should be acknowledged that the number of clinical cases examined by the physicians during the study period was sufficient to draw safe conclusions.

In conclusion, respiratory disorders mainly of the upper tract dominated among foreign travelers seeking medical services during vacations in Zakynthos island; the majority of these disorders were ear infections. Traveler's diarrhea was the most common specific diagnosis in this study.
